# Limitations and prospects in the management of IPMN: a retrospective, single-center observational study

**DOI:** 10.1186/s12893-023-01902-1

**Published:** 2023-01-07

**Authors:** Sarah Peisl, Oliver Burckhardt, Bernhard Egger

**Affiliations:** Department of Surgery, HFR Fribourg-Cantonal Hospital, Chemin des Pensionnats 2-6, 1708 Fribourg, Switzerland

**Keywords:** Pancreas, Intraductal papillary mucinous neoplasms, IPMN, Pancreatic cysts, Pancreatectomy

## Abstract

**Background:**

With increasing use and enhanced accuracy of cross-sectional imaging, the diagnosis of intraductal papillary mucinous neoplasms (IPMNs) of the pancreas has increased over the last few decades. The extent to which malignant transformation occurs remains unclear, making the management of IPMNs controversial. The aim of this study was to evaluate the progression rate and outcome of follow-up in patients with IPMNs.

**Methods:**

A database of all patients diagnosed with IPMN at the Cantonal Hospital HFR Fribourg, Switzerland, between January 2006 and December 2019 with a follow-up of at least 6 months was analyzed retrospectively. Descriptive statistics were performed on patient demographics, IPMN characteristics, and follow-up data.

**Results:**

A total of 56 patients were included in this study. Ten patients underwent primary surgery, 46 were enrolled in a surveillance program.21.7% (n = 5) of patients under surveillance presented with worrisome features of IPMN; progression rates were significantly higher in these patients (p = 0.043). Most progression occurred in the early follow-up period. Five patients underwent surgery due to progression, of which 2 presented high-grade dysplasia and 2 malignancy on postoperative histology.

**Conclusions:**

The limited predictive value of current guidelines may lead to surgical overtreatment, and the decision to proceed with surgical resection should be made with caution. Further prospective analyses and the development of novel biomarkers are needed to better understand the natural history of IPMN and improve diagnostic precision.

## Introduction

With increasing use and enhanced accuracy of computed tomography (CT) and magnetic resonance imaging (MRI), the incidental detection of intraductal papillary mucinous neoplasms (IPMNs) of the pancreas has increased over the last few decades. Up to 15% of patients undergoing abdominal MRI are diagnosed with a cystic lesion, which may be an IPMN in up to 82% of cases [1].

IPMNs of the pancreas are mucous-producing, cystic tumors originating from the pancreatic epithelium of the main pancreatic duct (MPD) or its side branches. Accordingly, they are classified into three groups [main duct (MD), branch duct (BD), or mixed type (MT) IPMN] and can evolve as unifocal or multifocal lesions. IPMN can be considered benign, borderline, or malignant depending on the grade of dysplasia [2–4]. Even though such lesions should always be considered as precursor lesions for pancreatic ductal adenocarcinoma (PDAC), it remains unclear the proportion to which malignant transformation occurs, with reported rates varying between 18 and 25% for BD-IPMN and 60% and 70% for MD-IPMN [5, 6]. To avoid malignant transformation, surgical resection remains the treatment of choice. However, the morbidity inherent with pancreatic surgery is not negligible and, as many IPMNs will never progress to malignancy, unnecessary surgery should be avoided [5, 6].

Due to the lack of knowledge regarding the natural history of these lesions, the management of IPMN remains controversial. Several guidelines based on expert opinions and retrospective studies have been established to predict the malignant potential and provide guidelines for follow-up and surgical decision-making ^3, 4, 7–9^. Guidelines developed by the International Association of Pancreatology [7] and the European guidelines from the European Study Group on Cystic Tumours of the Pancreas [8] are both widely used and identify obstructive jaundice, the presence of an enhancing mural nodule ≥ 5 mm or a solid cyst component, positive cytology for high-grade dysplasia (HGD) or invasive cancer, and a dilated MPD ≥ 10 mm as highly predictive of advanced neoplasia in IPMN and an indication for resection in surgically fit patients [9]. Further investigations, such as endoscopic ultrasound, should be performed in the presence of worrisome features [WFs; recurrent pancreatitis, cyst size ≥ 3 cm, cyst growth rate of ≥ 5 mm/2 years, thickened or enhancing cyst walls, MPD ≥ 5 mm, abrupt change in MPD caliber with distal pancreatic atrophy, and lymphadenopathy or increased serum level of carbohydrate antigen (CA19-9)], which are considered indications for surgery. Especially for MD- and MT-IPMN, a high index of suspicion is warranted due to higher risk of malignant transformation compared to BD-IPMN. For most patients with BD-IPMN, surveillance programs with periodic MRI every 6 to 24 months are recommended, with the interval of surveillance depending on the size of the largest cyst [3, 7, 8].

Current guidelines for the management of IPMN are based on limited evidence, and the safety, method, and duration of surveillance are still highly debated. Therefore, the aim of the present study was to analyze the progression rates and management strategies in all patients with IPMN who entered a surveillance program or were treated surgically at our center.

## Material and methods

### Study procedures

We conducted a retrospective observational study of patients presenting with an IPMN of the pancreas who were under surveillance or treated in the surgical clinic at the Cantonal Hospital HFR Fribourg, Switzerland. This study was approved by the cantonal Ethics Committee (Project-ID 2020-00332, Ethics Committee Vaud) and was conducted in accordance with the guidelines and regulations of the Declaration of Helsinki. The data were studied following the STROBE guidelines [10].

### Patient eligibility

We included all patients diagnosed with an IPMN of the pancreas within the surgical clinic at the Cantonal Hospital Fribourg, Switzerland, between January 2006 and December 2019. At the time of diagnosis, patients were either planned for primary surgical resection or enrolled in a surveillance program. Exclusion criteria were follow-up shorter than 6 months in patients enrolled in the surveillance program and misdiagnosis established radiologically during surveillance or histologically in the surgical specimen. Cases in which the diagnosis changed after initial diagnosis of IPMN were excluded in order to minimize selection bias. Informed consent was provided by all study participants.

### Data collection

All data were collected from the patient’s medical records. Random study ID allocation was generated via Excel® and data extraction performed using REDcap®. Extracted data included demographic data (age, sex, and comorbidities), radiographic reports from MRI, CT, endosonography (EUS) with or without fine-needle aspiration biopsy (FNA), and ultrasound, surgical and other physician consultations (gastroenterology, emergency room, and general practitioner), operative reports, and histological and cytological reports.

### Definitions

Patients were defined as being enrolled in the surveillance program if there was no surgical resection performed at time of diagnosis after the initial work-up. The surveillance program is supervised and led by the head pancreatic surgeon of our tertiary center and consists of a regular clinical and radiological follow-up according to current guidelines [3, 7, 8]. Follow-up duration was recorded as time in months between the initial IPMN diagnosis on first cross-sectional imaging and last available imaging data. WFs and high-risk stigmata (HRS) were defined according to criteria from current guidelines [3, 7, 8]. IPMN progression was defined as the appearance of at least one WF during follow-up.

### Statistical analysis

Medians with interquartile ranges (IQR), or percentages were calculated for the overall sample and subgroups. Descriptive statistics were computed for the patient’s demographics, mode of detection, and IPMN characteristics at diagnosis, as well as follow-up data and progression rates. Dichotomous data were reported as the number and proportions, continuous data as medians with interquartile range. Progression-free survival was estimated using the Kaplan–Meier method. Progression-free survival between the subgroups was compared by the Log rank test. All statistical analyses were performed using IBM® SPSS® Statistics 26. A two-sided level of significance of 0.05 was used for all analyses.

## Results

### Patient characteristics

**Fig. 1:**
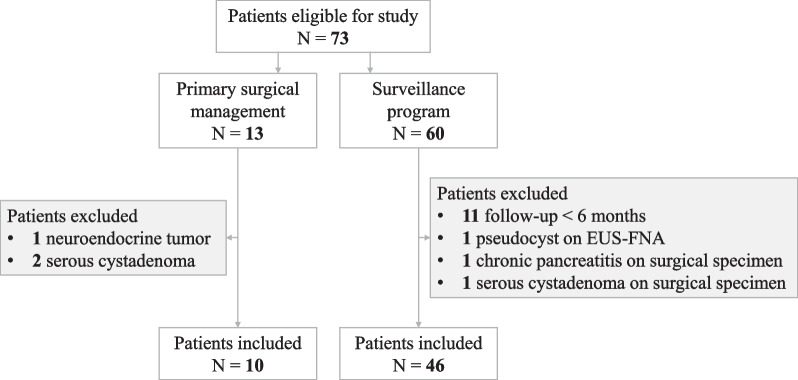
Flow chart of patient selection

Figure 1 illustrates patient selection. Between 2006 and 2019, 73 patients were diagnosed with an IPMN at our center. Among them, 13 were planned for primary resection, 3 of which were excluded because histological analysis of the surgical specimen concluded in another diagnosis (1 neuroendocrine tumor of the pancreas and 2 serous cystadenoma). Among the 60 patients initially selected for a surveillance program, 3 were excluded as diagnosis of IPMN was discarded during follow-up (1 pseudocyst diagnosed by EUS-FNA, 1 chronic pancreatitis with pseudocysts, and 1 serous cystadenoma on surgical specimen after secondary surgical resection) and 11 were excluded due to follow-up shorter than 6 months. We finally included 56 patients presenting with an IPMN of the pancreas at our center between 2006 and 2019, 10 of whom underwent primary surgery and 46 were enrolled in the surveillance program. Table 1 describes the characteristics of the study participants at diagnosis; 50.0% (n = 28) of the included patients were women, and the median age at diagnosis was 68 years (IQR = 9.5). Among the included patients, 16.1% (n = 9) presented with chronic pancreatitis and 14.3% (n = 8) with diabetes as a comorbidity. IPMN was diagnosed by abdominal CT in 82.2% (n = 46) of cases. In 21.4% (n = 12) of patients, abdominal pain or further imaging in the context of acute pancreatitis led to the diagnosis of IPMN. However, in most patients, IPMN was an incidental finding on imaging (78.6%, n = 44)). Diagnosis was then confirmed by MRI and / or EUS in all patients but three (93.5%, n = 43). These three patients did regular follow-up by CT due to colon or head and neck cancer.

**Table 1:** Characteristics of patients at diagnosis

	Overall (n = 56)
Male	28 (50.0)
Female	28 (50.0)
Median age at diagnosis (IQR) [years]	68 (9.5)
Comorbidities	
Chronic pancreatitis	9 (16.1)
Diabetes	8 (14.3)
Detection	
Incidental finding	44 (78.6)
Abdominal pain	7 (12.5)
Pancreatitis	5 (8.9)
Mode of detection	
CT	46 (82.2)
MRI	7 (12.5)
US	3 (5.4)
BD-IPMN	41 (73.2)
MD-IPMN	9 (16.1)
MT-IPMN	6 (10.7)
Localization of IPMN^1^	
Head	22 (39.3)
Body	15 (26.8)
Uncinated process	14 (25.0)
Tail	9 (16.1)
Neck	8 (12.3)
Median cyst size at diagnosis (IQR) [mm]	16.0 (13)
MPD-size 3.5–5 mm	10 (17.9)
WF at diagnosis (WF +) ^2^	15 (26.8)
Cyst size ≥ 3 cm	9
Thickened or enhancing cyst walls	2
MPD-size 5–9 mm	8
Recurrent pancreatitis	2
HRS at diagnosis (HRS +)^2^	3 (5.6)
Obstructive jaundice	1
High-grade dysplasia on EUS-FNA	2

Values are n (%) unless otherwise noted. *BD* branch duct, *MD* main duct, *MT* mixed type, *WF* worrisome feature, *MPD* main pancreatic duct, *IQR* interquartile range

^1^Multiple localizations possible

^2^Presence of at least one worrisome feature or one high-risk stigmata at diagnosis. Multiple possible

The most frequent IPMN types at diagnosis were BD-IPMN (73.2%, n = 41), followed by MD-IPMN (16.1%, n = 9) and MT-IPMN (10.7%, n = 6); 39.3% (n = 22) of the IPMNs were localized in the head of the pancreas, 26.8% (n = 15) in the body, 25.0% (n = 14) in the uncinated process, 12.3% (n = 8) in the neck, and 16.1% (n = 9) in the pancreatic tail. Median cyst size at diagnosis was 16 mm (IQR = 13). Overall, 26.8% (n = 15) of patients presented with WFs at diagnosis, including 9 patients diagnosed with cyst ≥ 3 cm. Three patients presented with HRS at diagnosis (5.6%) and underwent primary surgery without previous enrollment in the surveillance program.

### Surveillance program

Forty-six patients were enrolled in the surveillance program (Table 2).

**Table 2:** Follow-up of patients enrolled in the surveillance program

	Overall (n = 46)	WF at diagnosis WF + (n = 10)	No WF at diagnosis WF− (n = 36)
BD-IPMN	36 (78.3)	5 (50.0)	31 (86.1)
MD-IPMN	7 (15.2)	3 (30.0)	4 (11.1)
MT-IPMN	3 (6.5)	2 (20.0)	1 (2.8)
Diagnosis confirmed by MRI and / or EUS	43 (93.5)	10 (100)	3 (83.3)
Diagnostic EUS-FNA	13 (28.3)	5 (50.0)	8 (22.2)
EUS-FNA during surveillance	17 (37.0	6 (60.0)	11 (30.6)
Median follow-up time (IQR) [months]	23 (27)	21 (12.8)	23 (27.5)
Median time to progression (IQR) [months]	11 (9)	16 (5)	9 (4)
Progression during FU (appearance of new WF)^1^	11 (23.9)	5 (50.0)	6 (16.7)
Cyst size ≥ 3 cm	2	0	2
Cyst growth rate of ≥ 5 mm/2 years	9	5	4
Thickened or enhancing cyst walls	4	1	3
MPD-size ≥ 5 mm	1	0	1
High-grade dysplasia on EUS-FNA	0	0	0
Surgery	5 (10.9)	2 (20.0)	3 (8.3)
IPMN progression (rapid cyst growth)	3	2	1
Concomitant disease	2	0	2
Surveillance status			
Active surveillance	25 (54.4)	4 (40.0)	21 (58.3)
FU stopped (comorbidities)	2 (4.3)	1 (10.0)	1 (2.8)
FU stopped (death)	1 (2.2)	0 (0.0)	1 (2.8)
FU stopped (regression, disease stability)	2 (4.3)	1 (10.0)	1 (2.8)
Patient did not wish to continue FU	9 (19.6)	2 (20.0)	7 (19.4)
Lost to follow-up	2 (4.3)	0 (0.0)	2 (5.6)

Values are n (%) unless otherwise noted. BD: branch duct, MD: main duct, MT; mixed type, WF: worrisome feature, MPD: main pancreatic duct, FU: follow-up, EUS-FNA: endoscopic ultrasound-guided fine-needle aspiration, IQR = interquartile range

#### Patients with WF at diagnosis (WF +)

Ten patients presented with an IPMN with WFs at diagnosis (21.7%). The median follow-up time was 21 months (IQR = 12.8) with a median time to progression of 16 months (IQR = 5). Half of the patients underwent diagnostic EUS-FNA, and further EUS-FNA was performed during follow-up in 60% (n = 6) of the cases. Of all patients with WF + IPMN, 50% (n = 5) showed progression during surveillance and 20% (n = 2) underwent surgery.

Eight patients had a single WF (cyst size ≥ 3 cm, MPD dilatation ≥ 5 mm or enhancing cyst walls). Three of these patients exhibited progression after 10, 16, and 19 months: One patient underwent surgery (#12 in Table 3), one patient decided to pursue surveillance, and one patient refused further investigations and surveillance. Two patients presented with more than one WF. In the first case, explorative laparotomy with the objective of primary resection by partial pancreatectomy was performed in a 70-year-old female patient 9 months after first diagnosis of a multifocal MT-IPMN of the entire pancreas with a maximum cyst size of 36 mm and MPD dilatation of 11 mm. As the patient refused total pancreatectomy, which was indicated due to the extent of the disease, resection was not performed and she was enrolled in the surveillance program. Cyst growth of 9 mm/2 years (to 45 mm) was noted during 71 months of surveillance, and then disease stability for another 50 months, bringing the total follow-up duration up to 121 months. In a second case, a 50-year-old male with MT-IPMN 84 mm in size with MPD dilatation of 8 mm was enrolled in the surveillance program due to normal results on cytology and normal serum CA19-9 levels. However, due to progression after 14 months (cyst size 100 mm), the patient (#11 in Table 3) underwent pancreaticoduodenectomy 23 months after diagnosis, revealing HGD.

**Table 3:** Detailed characteristics of patients who underwent pancreatic surgery

	ID	Age [years]	ASA-Score	Sex	Radiological diagnosis	HRS at diagnosis	WF at diagnosis	Time from diagnosis [months]	Indication for surgery	Procedure*	Postoperative histopathological diagnosis
Primary surgery	#1	72	2	M	MT, head, cyst 10 mm, MPD 5 mm	−	** + **	13	WF at diagnosis (recurrent pancreatitis + MPD dilatation)	DP	MT-IPMN, 20 mm, gastric type, moderate dysplasia
	#2	65	2	F	BD, head/neck, cyst 55 mm	−	** + **	3	WF at diagnosis (cyst size + enhancing cyst walls)	PD	MT-IPMN, 46 mm, gastric type with PDAC pT1a (2 mm) pN0 (0/14) LV0 Pn0 R0
	#3	61	2	F	MT, tail, cyst 16 mm, MPD 6 mm	−	** + **	9	WF at diagnosis (MPD dilatation)	DP	BD-IPMN, 20 mm, gastric type, low-grade dysplasia
	#4	78	3	M	MD, tail, cyst 30 mm	−	** + **	7	WF at diagnosis (recurrent pancreatitis + cyst size)	DP	MD-IPMN, type unknown,low-grade dysplasia
	#5	71	2	M	BD, head, cyst 13 mm	** + **	** + **	2	HRS at diagnosis (HGD at EUS-FNA)	PD	BD-IPMN, 14 mm, gastric type, low-grade dysplasia
	#6	51	2	M	BD, uncinate process, cyst 18 mm	** + **	** + **	3	HRS at diagnosis (HGD at EUS-FNA)	PD	BD-IPMN, 18 mm, gastric type, low-grade dysplasia
	#7	65	2	F	MT, head, cyst 28 mm, MPD 6 mm	−	** + **	1	HRS at diagnosis (MPD dilatation)	PD	MT-IPMN, 20 mm, gastric type, moderate dysplasia
	#8	75	2	M	MD, head, cyst 29 mm	** + **	** + **	1	HRS at diagnosis (obstructive jaundice + cyst size)	TP	MT-IPMN, gastric type with PDAC pT3 (38 mm) pN1 (3/18) LV0 pN1 R0
	#9	71	2	M	BD, tail, cyst 12 mm	−	−	3	Concomitant NET of the pancreatic tail	DP	BD-IPMN, 15 mm, gastric type, no dysplasia (NET, 6 mm, G1)
	#10	53	2	F	BD, tail, cyst 14 mm	−	−	2	Elevated cyst CEA (suspicion mucinous cystadenoma)	DP	BD-IPMN, 14 mm, type unknown, low-grade dysplasia
Surveillance program	#11	50	3	M	MT, head, cyst 84 mm, MPD 8 mm	−	** + **	23	Progression (cyst size 100 mm, rapid cyst growth)	PD	MT-IPMN, 100 mm, oncycytic type withhigh-grade dysplasia
	#12	63	3	M	MD, head, cyst 19 mm, MPD 6 mm	−	** + **	19	Progression (rapid cyst growth)	PD	MD-IPMN, intestinal type high-grade dysplasia
	#13	56	2	M	BD, uncinate process, cyst 20 mm	−	−	10	Progression (cyst size 30 mm, rapid cyst growth)	PD	BD-IPMN, gastric type low-grade dysplasia
	#14	67	2	F	BD, neck/body, cyst 26 mm	−	−	45	Concomitant serous cystadenoma	PD	BD-IPMN, 15 mm, pancreatico-biliary type, low-grade dysplasia (serous cystadenoma, 11 mm)
	#15	64	3	M	BD, tail, cyst 17 mm	−	−	15	Concomitant gastric GIST	DP + gastric resection	BD-IPMN, 20 mm, gastric type, low-grade dysplasia (gastric GIST, 35 mm)

*HRS* High-risk stigmata, *WF* worrisome features, *BD* branch duct, *MD* main duct, *MT* mixed type, *MPD* main pancreatic duct, *NET* neuroendocrine tumor, *GIST* gastrointestinal stromal tumor, *PDAC* pancreatic ductal adenocarcinoma, *PD* pancreaticoduodenectomy, *DP* distal pancreatectomy, *CP* central pancreatectomy, *TP* total pancreatectomy, *EUS-FNA* endoscopic ultrasound-guided fine-needle aspiration, *HGD* high-grade dysplasia, *CEA* carcinoembryonic antigen

^*^All surgical resections were performed with standard lymphadenectomy according to the ISGPS consensus [51]

#### Patients without WF at diagnosis (WF−)

Thirty-six (78.2%) patients in the surveillance program did not exhibit WFs at diagnosis. Six of these patients presented progression during follow-up (16.7%), with a median time to progression of 9 months (IQR = 4). The median follow-up time was 23 months (IQR = 27.5). Eight patients (22.2%) underwent diagnostic EUS-FNA and further EUS-FNA was performed during the course of surveillance in 30.6% of the cases.

One patient (#13 in Table 3) underwent surgery after 10 months of follow-up due to rapid cyst growth in a young, fit, male patient with BD-IPMN (see Table 3). Two patients presented with concomitant disease, which led to the indication for surgery (serous cystadenoma of the pancreas in patient #14 and gastric GIST in patient #15, which was also treated by gastric wedge resection during the intervention).

#### Overall progression

**Fig. 2:**
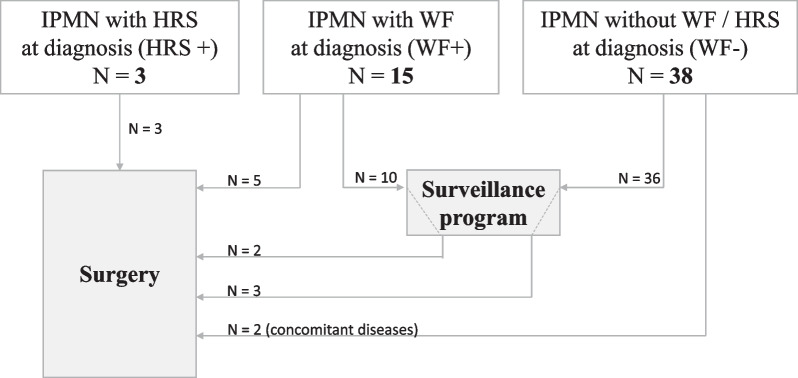
Flow chart of clinical management

Of the 46 patients who entered surveillance, 5 underwent surgical resection. Progression during follow-up led to surgical management in three of the patients, and concomitant disease led to surgery in two of the patients (see Fig. 2).

**Fig. 3:**
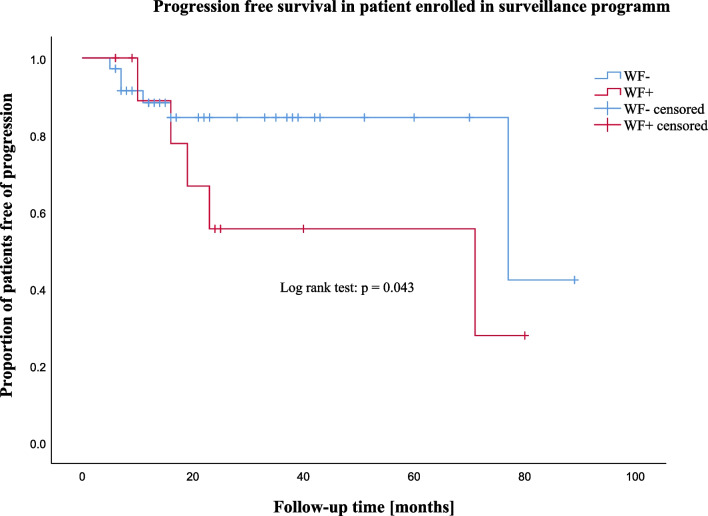
Kaplan–Meier curve of progression-free survival in patients enrolled in the surveillance program

Figure 3 shows the progression-free survival for patients enrolled in the surveillance program. Most new WFs appeared during early follow-up, but some progression occurred after up to 77 months. Median time to progression in patient with WFs at diagnosis (WF +) was 16 months (IQR = 5) versus 9 months (IQR = 4) in patients presenting with no WF at diagnosis (WF-). There was a significantly higher rate of progression-free survival during follow-up in WF- patients compared to WF + patients (p = 0.043).

In two patients, surveillance was stopped due to disease stability after 100 months or regression after 6 months. Overall, nine patients did not wish to continue follow-up (19.6%) and three of the patients (6.5%) withdrew from surveillance due to comorbidities and malignancies or death not related to IPMN.

### Primary surgical management

As shown in Table 3, 10 of the 56 included patients underwent primary surgical resection (17.9%). In three cases, patients presented with HRS (either HGD on cytology or obstructive jaundice) at diagnosis. In five cases, the indication for primary surgery was the presence of at least two WFs at diagnosis or one WF in MT-IPMN. One patient underwent surgical resection because of a concomitant neuroendocrine tumor of the pancreas and one patient due to elevated carcinoembryonic antigen (CEA) in the cyst fluid suspicious of mucinous cystadenoma. See Table 3 for details on surgically managed patients.

### Cytological and histological analyses

Preoperative EUS-FNA was performed in all patients undergoing surgical resection. Post-operative histological analysis of all patients undergoing surgery showed low-grade dysplasia in most patients (53.3%, n = 8). In the 10 patients undergoing primary surgery, 2 exhibited HGD preoperatively, but it was not confirmed on post-operative histological analysis. Malignant transformation was diagnosed in two patients who underwent primary surgery but did not exhibit HGD on preoperative cytology. In the five patients enrolled in the surveillance program, histological analysis of the surgical specimen found HGD in two cases but no cases of malignant transformation. Table 3 provides the details of the patients who underwent surgical resection.

### Surgical morbidity and mortality

Among the 15 operated patients, postoperative morbidity within 30 days after surgery was 33% (n = 5), with 2 presenting a complication grade II, 2 presenting a grade IIIa, and 1 patient presenting a grade IIIb according to Clavien-Dindo Classification [11]. At 1-year follow-up, postoperative mortality was found to be 0%. One patient (#5 in Table 3) died 16 months after surgery of a cause unrelated to surgery and one (#13 in Table 3) 7 years later of pancreatic adenocarcinoma that developed after 5 years of uneventful follow-up.

## Discussion

The primary goal in the management of patients with IPMN is to prevent progression to malignancy while avoiding unnecessary surgery. However, due to a lack of knowledge about the rate and timing of malignant progression, the management of this entity remains challenging even though the diagnosis of IPMN of the pancreas is increasing due to the increased use of advanced imaging [9, 12].

In accordance with the literature, our analyses showed that IPMNs are often an incidental finding, with at least 78.6% of patients in our series being asymptomatic. In patients enrolled in the surveillance program, 21.7% presented with WFs at diagnosis. In our series, these patients were shown to be significantly more likely to progress, suggesting the need for shorter follow-up intervals. Most patients showed progression early during surveillance but, in some cases, new WFs appeared after more than 5 years of disease stability. Accordingly, several authors have reported long-lasting risk of the development of concomitant PDAC in patients with IPMN, and even the risk of developing new IPMN after resection in up to 62% at 10 years, with potential malignant progression [1, 7, 13–16]. Therefore, indefinite surveillance is suggested, even after surgical resection of IPMN. However, some authors have recommend the discontinuation of follow-up after 2–5 years of disease stability due to the small overall risk of malignant transformation, which outweighs the cost of surveillance and the psychological burden of disease [17–19]. The required duration of surveillance remains debatable. In our series, 19.6% of the patients discontinued surveillance on their own initiative, suggesting significant psychological burden. The potential benefit from longer surveillance needs to be balanced with psychological burden and cost-effectiveness, as well as the potential benefit from surgical resection.

Surgery is widely accepted as the only curative treatment for IPMN. However, the morbidity and mortality associated with pancreatic surgery is high, with post-operative morbidity of up to 30–50% [20], and needs to be cautiously balanced with the potential risk of malignant transformation. The malignant potential of IPMN remains uncertain, ranging from 0 to 32%, with an estimated average annual risk of 0.24% in absence of HRS [17, 21] and differing between the different types of IPMNs, with increased risk in MD- and MT-IPMN compared to BD-IPMN [3, 7, 22, 23]. Different research groups have attempted to define WFs to predict the risk of malignancy [4, 7, 13, 17–19, 21, 24–27]. Some authors have described cyst size as the main predictor, increasing the risk of malignant transformation threefold in the presence of a cyst larger than 3 cm, and eightfold if a solid component is associated [4, 21, 28, 29]. Other authors have suggested that the presence of enhancing mural nodules is highly suspicious for malignant transformation [30–33]. Cytological analyses and serum tumor markers have been described as helpful tools in the risk-stratification of cystic lesions [3, 19, 24, 25, 32], but some authors have found no evidence to suggest this as a routine diagnostic method [26, 34]. In our study, cytological analyses by EUS-FNA were performed in all patients undergoing surgery and in 37% of the patients during surveillance. HGD was found in two patients who then underwent primary surgery. However, both surgical specimens only showed low-grade dysplasia. On the other hand, patients with HGD or malignancy in the surgical specimen did not present with HGD in the cyst fluid analysis, suggesting low predictive power as a single predictor.

Even though the different high-risk features seem to be associated with progression and malignant transformation, these features alone have a relatively poor positive predictive value, ranging from 25 to 62%, which may result in surgical overtreatment [4, 16, 30, 32, 35–38]. In our study, 12 patients underwent surgical resection for the presence of HRS or progression of WF. Of these patients, 2 presented HGD and 2 PDAC. This is in accordance with a recent series, which showed 10% of PDAC and 20% of HGD in patients after pancreatectomy for IPMN [38].

Definitive diagnosis can only be established pathologically in patients who have undergone resection. In our cohort, five patients underwent surgery for preoperative diagnosis of IPMN, which was not later confirmed to be an IPMN, but another cystic lesion of the pancreas, such as serous cystadenoma. In patients entering surveillance programs without surgical resection, IPMNs are diagnosed radiologically, which could lead to a significant amount of misdiagnosis of both presumed IPMN and the presumed absence of associated malignancy. It is impossible to evaluate the actual incidence of malignancy in the overall population of individuals harboring high-risk IPMNs.

New predictive factors are needed to more accurately predict malignancy and improve surgical decision-making. Recent studies have shown promising results with the analysis of cyst fluids [39]. IPMNs with low-grade dysplasia and HGD have distinct molecular features with, for example, different levels of prostaglandin E synthase 2 or interleukin 1β, which could be indicators of malignant progression and, therefore, serve as biomarkers to identify patients with high-risk IPMNs [40–42]. Furthermore, mutational analysis of driver genes, such as *KRAS*, *GNAS*, or *KLF4,* as well as miRNA sequencing and cyst fluid telomere fusion status seem to allow the discrimination of high-risk IPMN from low risk lesions [43–47]. Panel analyses combining several markers have shown to further increase the specificity and sensitivity to accurately predict high malignant potential [48–50].

Currently, the management of IPMNs is still highly debated, and risk factors for IPMN need to be more clearly defined according to molecular, radiological, and clinical data. Our study gives insights into IPMN follow-up and associated problems with risk stratification for these lesions. There are some limitations to this study, namely the small sample size and lack of information on the post-operative follow-up of our patients. Furthermore, not all IPMNs included in the analysis were histologically proven because they were not all resected. There is a clear need for prospective (multi-institutional) studies of the long-term follow-up of IPMNs and the analysis of both molecular and clinical data.

## Conclusion

In conclusion, IPMNs are a frequent entity with increasing incidence. Evidence-based guidelines regarding clinical management are lacking. Even though the presence of worrisome features seems to be associated with higher risk of progression, its prediction for the risk of malignancy remains uncertain. The limited positive predictive value of current guidelines may lead to surgical overtreatment, and the decision to intervene surgically should be made with precaution. Clinical decision-making should be based on the estimated risk of malignant transformation, patient’s age, comorbidities, and life circumstances. Further prospective analyses and the development of novel biomarkers are needed to better understand the natural history of IPMNs and improve diagnostic precision. In the meantime, the complexity of the management of IPMN should not be underestimated and should be centralized in centers with high volume and expertise.

## Data Availability

All data generated or analyzed during this study are included in this published article.

## Abbreviations

CT: Computed tomography
IPMN: Intraductal papillary mucinous neoplasms
MPD: Main pancreatic duct
MD: Main duct
BD: Branch duct
MT: Mixed type
PDAC: Pancreatic ductal adenocarcinoma
CA 19-9: Carbohydrate antigen
HGD: High-grade dysplasia
WF: Worrisome feature
EUS: Endosonography
FNA: Fine-needle aspiration biopsy
HRS: High-risk stigmata
CEA: Carcinoembryonic antigen
IQR: Interquartile range
